# Scaling trends of bird’s alular feathers in connection to leading-edge vortex flow over hand-wing

**DOI:** 10.1038/s41598-020-63181-7

**Published:** 2020-05-13

**Authors:** Thomas Linehan, Kamran Mohseni

**Affiliations:** 1 Department of Mechanical & Aerospace Engineering, University of Florida, Florida, 32611 USA; 2 Department of Electrical and Computer Engineering, University of Florida, Florida, 32611 USA

**Keywords:** Fluid dynamics, Biomechanics, Aerospace engineering

## Abstract

An aerodynamic structure ubiquitous in Aves is the alula; a small collection of feathers muscularized near the wrist joint. New research into the aerodynamics of this structure suggests that its primary function is to induce leading-edge vortex (LEV) flow over bird’s outer hand-wing to enhance wing lift when manuevering at slow speeds. Here, we explore scaling trends of the alula’s spanwise position and its connection to this function. Specifically, we test the hypothesis that the relative distance of the alula from the wing tip is that which maximizes LEV-lift when the wing is spread and operated in a deep-stall flight condition. To test this, we perform experiments on model wings in a wind tunnel to approximate this distance and compare our results to positional measurements of the alula on spread-wing specimens. We found the position of the alula on non-aquatic birds selected for alula optimization to be located at or near the lift-maximizing position predicted by wind tunnel experiments. These findings shed new light on avian wing anatomy and the role of unconventional aerodynamics in shaping it.

## Introduction

**Figure 1:**
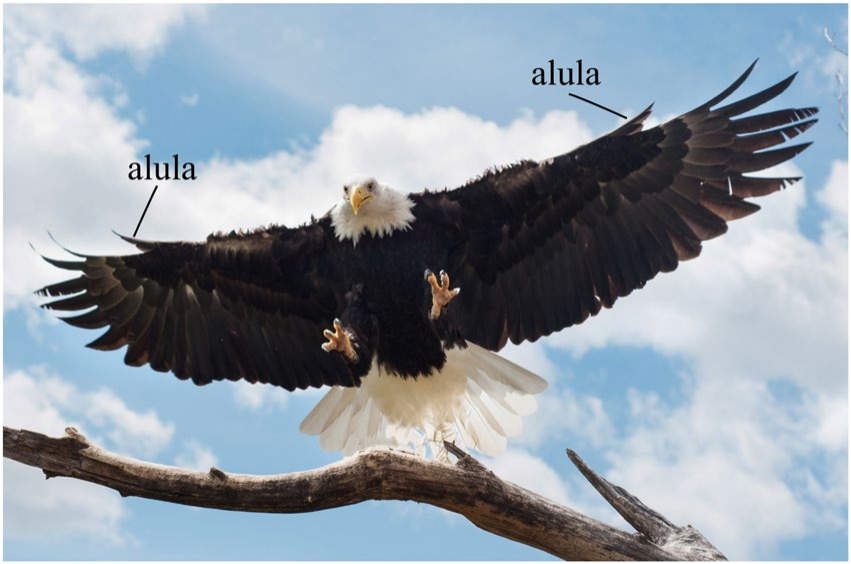
Spread-wing gliding posture used by birds to airbrake when executing a glide-assisted landing. Deflected lesser covert feathers implicate that the wings are operated at a deep-stalled flight condition. Protracted alula is labeled. Image taken by Kathleen Sue Sullivan.

A bird’s alula consists of a small cohort of feathers, approximately one-eighth the length of the bird’s wing, that stem from the bird’s primary digit, or thumb. It is an evolutionary adaptation observed in fossils of primitive birds^1–3^, and exists on all modern birds (minus hummingbirds)^4^. The function of the alula is widely considered to be aerodynamic, although some research has indicated a possible sensory role^5^. During landing, birds tilt their wings to high angles to slow descent^4,6^ and protract their alula upwards from the plane of the wing^6^ to prevent wing stall and the subsequent loss of wing lift^7–14^ (see Fig. 1). This function enables birds to perform steeper descents with greater changes in body orientation when landing^10^. Thus, additional knowledge of these flight feathers can advance our understanding of avian flight.

### Aerodynamics of the alula

Despite consensus among researchers regarding the importance of the alula in avian flight, the aerodynamic mechanisms underlying its function remain debated. The gap formed between the deflected alula and the top surface of the wing (see Fig. 1) has led to early comparisons of it to flow control devices on aircraft such as leading-edge slots/slats^7–9^. These devices prevent wing stall by ensuring the flow remains smoothly attached to the wing. Subsequent research depicting separated flow over real and model swift wings in steady flight^15,16^ and Passerines in slow-flapping flight^17^ suggests that the alula likely prevents wing stall through the maintenance of separated-edge flows rather than preventing flow separation from occurring in the first place.

These observations have prompted a revaluation of the aerodynamics of the alula for which two updated interpretations of its function have been proposed^4,6^. First, that the alula generates a small vortex which separates the attached-flow system on the inner, thick-profiled, arm-wing section and the separated leading-edge vortex (LEV) on the outer, thin-profiled, hand-wing section. This function has been partially corroborated by several recent experimental and computational works. Using planar PIV, Lee *et al*.^10^ measured a streamwise vortex aft of the alula, calling this the alula tip vortex, and a stall-delaying effect outboard of the alula. Sander^18^ also observed an alula tip vortex as well as an alula leading-edge vortex in their computations on a simplified bird-alula model. However, Lee *et al*. did not measure nor mention the LEV on the hand-wing and Sander only observed an LEV when simulating flapping motion. Sander notes the breakdown of the alula vortices at higher angles of attack, *α* ≥ 25 deg, due to their interaction with a separated wing boundary layer. These observations led Sander to remark that the streamwise vortex measured by Lee *et al*.^10^ is likely not the alula tip vortex but rather the separated boundary layer of the alula.

**Figure 2:**
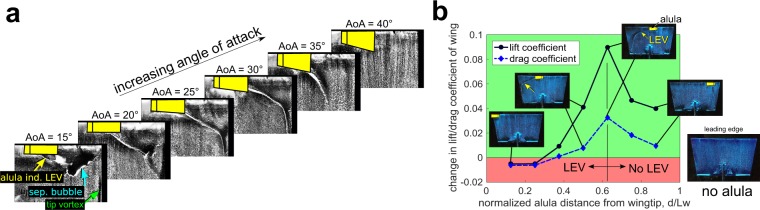
Model alula induces apparent leading-edge vortex (LEV) with the alula’s distance from the wing tip controlling LEV lift. (**a**) Effect of angle of attack on the near surface flow structures outboard of the alula. The stall angle of the wing with no alula is approximately 22 deg. (**b**) Change in lift and drag coefficient relative to wing without an alula as a function of the alula’s root distance from the wing tip normalized by wing length. Inset figures are corresponding images of oil-patterns at labeled data points. Arrows indicate surface-footprint of apparent alula-induced LEV. Lift and drag increment rapidly increases as the alula is distanced further from the wing tip until drastically decreasing for alula distances for which the LEV is lost.  = 1.5 wing is inclined to steady flow at angle of attack of 25 deg, ${C}_{L}=0.73$. The peak increase in lift coefficient due to the alula is approximately 13%. Figures adapted from Linehan and Mohseni^19^.

A second proposed function of the alula is that it promotes LEV formation over the swept-back hand-wing of birds in flight scenarios when the arm-wing is completely stalled^4,6^. Carruthers *et al*.^6^ observed the steppe eagle *Aquila nipalensis* to morph its wings into a distinct M-shape during the pitch-up phase of its perching sequence. They hypothesized the alula in this scenario to operate like a strake on a delta-winged aircraft, producing a vortex that promotes LEV formation on the swept-back hand wing. The simulations of Sander^18^ showed the LEV on the swept-back outer wing only during flapping simulations involving a wing both with, and without, the alula. Sander noted negligible changes in the flow topology and pressure distribution between cases and further observed the alula tip vortex in this scenario to be sucked into the stronger LEV. The first qualitative experimental evidence of an alula stabilizing an LEV was shown by Linehan and Mohseni^19^ in an exploratory investigation into the aerodynamics of a model alula affixed to a thin-profiled rectangular wing. Surface-oil visualizations conducted at a range of angles of attack encompassing pre-stall and post-stall angles, revealed an apparent LEV outboard of the alula (see Fig. 2a). This vortex was observed to interact with separation bubble-type flow at pre-stall angles of attack causing a reduction in wing lift. In contrast, lift-enhancement of the alula at post-stall angles was tied to the ability of the LEV to sweep across the outer edges of the wing. Their findings of an apparent alula-induced LEV on an unswept wing indicates that wing sweep is not necessary for with stability. In the current work, we confirm the existence of this flow pattern on an unswept wing, quantitatively, by directly measuring the flow over a model wing with an alula using stereoscopic-digital particle image velocimetry. From these findings, we then reevaluate the role of the swept-back hand-wing in the landing flight of birds in the Discussion section of this work.

We conclude from the above recount that two aerodynamic mechanisms of the alula exist depending on the status of wing flow (weakly separated or massively separated). The aerodynamic mechanism of the alula when the wing is weakly separated is likely associated with the interaction of alula vortices (alula tip vortex and alula leading-edge vortex) and a separation bubble over the wing. Whereas the aerodynamic mechanism of the alula when the wing is massively separated is associated with its ability to induce and stabilize an LEV on the outer portion of the wing. The latter mechanism appears to be the critical one as the ability to induce LEV flow over a wing in a deep-stall condition would enable birds to tilt their wings to extreme angles of attack to increase drag while sustaining an ability to adjust attitude via alula-induced LEV flow. This would be particularly beneficial to assist maneuvering and landing flight in cluttered environments. Furthermore, the finding that a sweptback outer wing is not necessary for the stability of the alula-induced LEV suggests that aerodynamic forces can be maximized by allowing the bird to assume a spread-wing gliding posture while still leveraging LEV-lift for enhanced flight control. Indeed, force measurements of real swift wings in a wind tunnel performed by Lentink *et al*.^16^ show that swept wings always generated less lift than extended (or spread) wings, noting that the extra lift from LEVs (stabilized via wing sweep) does not compensate for lift lost due to the simultaneous drop in wing area and aspect ratio.

### A lift-maximizing spanwise position of the alula

Considering the importance (and ubiquity) of the alula in bird flight, one may expect the design of these flight feathers to be optimized in a manner that takes advantage of their aforementioned aerodynamics. Several exploratory investigations into the aerodynamics of the alula on model wings have found the spanwise position of the alula to influence post-stall wing lift^13,14,19^. The effect of the alula’s spanwise position specifically on alula-induced LEV lift was noted in Linehan and Mohseni^19^ for which relevant results are reproduced in Fig. 2b. LEV-lift is observed to rapidly increase as the alula is distanced further from the wing tip while drastically decreasing for alula distances for which the LEV flow pattern is lost. These results depict a strong sensitivity in the alula’s spanwise position on the wing in terms of its lift benefit, where small deviations from the lift-maximizing value result in steep performance losses. In this manuscript, we test the hypothesis that the (fixed) relative location of birds alular feathers is that which maximizes the lift-benefit of the alula in the aforementioned manner.

### Scaling trends in flight feathering

Measurements of the alular feathers are sparse in the literature^7,10,11^. Among this work, the authors are only aware of one study that reported positional measurements of the alula across species of varying taxa. Alvarez *et al*.^7^ measured parameters of the alula and wing on 40 bird species from Spain and showed that the alula’s relative spanwise position on the wing was dependent on wing shape and therefore flight style. The alula’s distance from the wing tip on birds categorized with elliptical-type wings was found to range from 0.7–0.75 times the wing length. Birds categorized with high-speed wings were found to have the alula stationed closer to the wing root while birds with broad-type high-lift wings tended to have the alula stationed closer toward the wing tip. However, the latter categories of wing shape are not well represented in the data set and additional measurements are needed to clarify these trends.

Trends in the alula’s relative spanwise position on the wing can also be deciphered by considering the anatomy of bird’s wings and the organization of flight feathers. The alula bridges the gap between two distinguished parts of the avian wing^4^. Inboard of the alula, is the bird’s thick-profiled arm-wing region layered with covert feathers and internally composed of arm bones such the humerus and ulna. Outboard of the alula is the bird’s thin-profiled hand-wing region, comprised of the greater primary coverts and the primary feathers. From these insights, we expect scaling trends of the alula’s position to reflect that observed for covert feather extent and primary feather length. Wang and Clarke^20^ used a geometric-morphometric approach to describe the wing outline as well as the extent of dorsal and ventral covert feathers on 105 avian species. They found an elongation of covert feathers in taxa with aquatic ecologies (despite differences in foraging behavior and flight style) and found Passerines to have the shortest coverts. They remarked that the “Elongation of covert feathers in taxa with aquatic ecologies could be related to behavioral modifications linked to this ecology (e.g. take off from the water surface or take off angle), but the reason for such elongation remains wholly uninvestigated”. The alula’s distance ratio is more directly related to the relative length of bird’s primary feathers. From an analysis of arm bone length (humerus + ulna + manus, ${t}_{a}$) of 748 extant bird species, Nudds^21^ predicted the scaling of primary feather length to wingspan to be ${f}_{prim}\propto {b}^{0.93}$. In recovering this scaling, Nudds assumed ${t}_{a}+{f}_{prim}\propto {b}^{1}$ implying a constant elbow angle across bird species. Nudds^22^ then directly measured primary feather length and arm bone length on 34 species which did not disprove this scaling. As stated by Nudds^22^, “Although tentative at this stage, the scaling of $ta$ and ${f}_{prim}$ may be the product of an as yet unidentified ratio for feathers to wing-skeleton length within the biomechanical and aerodynamic constraints acting upon the scaling of $b({M}^{\mathrm{1/3}})$.” A deeper understanding of the unconventional flight mechanisms of birds in relation to avian wing and feather design may help to explain these trends.

### Current approach

The goal of this study is to test the hypothesis that the relative spanwise location of bird’s alula is that which maximizes LEV-lift when birds spread their wings to airbrake as necessary for maneuvering or landing in cluttered environments. Towards this end, we first quantitatively confirm the existence of an alula-induced LEV on an unswept wing via flow measurements in a wind tunnel. Then, we analyze scaling trends of the alula’s position considering new positional measurements of the alula performed on spread-wing specimens. In our analysis of the bird data, we distinguish birds with aquatic and non-aquatic ecologies based on previous findings of the influence of these character traits on covert feather extent. Furthermore, we distinguish birds with differing wing shape and flight style based on prior research exposing the influence of these character traits on the relative alula position. Thereafter, we approximate the lift-maximal distance ratio on representative model bird wings via wind tunnel experiments and compare wind tunnel predictions to parameter estimates of the relative alula position measured on bird specimens. We then discuss our results, the limitations of the study, and potential future directions.

## Results

### Flow measurements of alula-induced LEV on model wing

**Figure 3:**
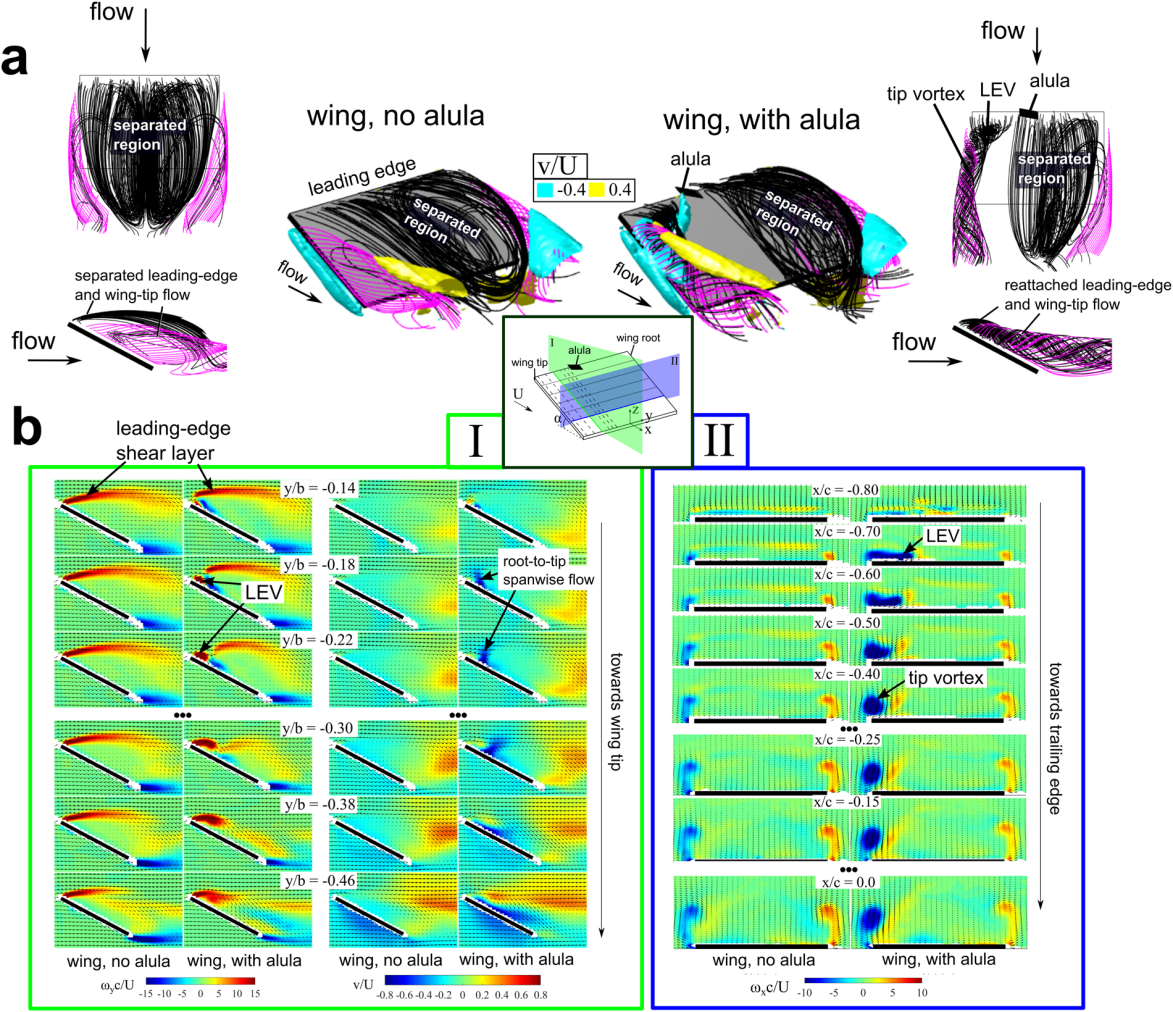
Model alula induces and stabilizes leading-edge vortex (LEV) over outer portion of unswept wing in deep-stall flight condition. (**a**) Three-dimensional streamlines of time-averaged flow measured around a model wing with and without the alula. Streamlines originating at the wing’s leading-edge are colored black. Streamlines originating at the wing’s tip are colored magenta. Isosurfaces of spanwise flow are included in isometric views.  = 1.5 wing at an angle of attack of 28 deg. Alula’s root is centered on the wing. (**b**) Corresponding contour slices of measured flow quantities. (I) Non-dimensional spanwise vorticity ${\omega }_{y}$ and velocity $v$ in streamwise-oriented planes at spanwise stations outboard of the alula. (II) Non-dimensional streamwise vorticity ${\omega }_{x}$ in spanwise-oriented planes along the chord of the wing. $c$ is the wing chord length, $b$ is the wing span, $U$ is the free-stream velocity, and $\alpha$ is the angle of attack.

To confirm the existence of an alula-induced LEV, we directly measured the global time-averaged flow-field over a model wing with an alula in a wind tunnel using a technique called stereoscopic-digital particle image velocimetry. In this experiment, we are modeling the wing-alula interaction on the half-wing of a bird in steady translation whose wings are spread and operated in a deep-stall condition (see Fig. 1). A rudimentary emulation of the alula-wing interaction was chosen as it isolates the essential physics underlying the alula’s aerodynamic function in the deep-stall flight condition, which may have been veiled if the more complex features of the alula and wing were included, but not properly modeled. The flight condition of a bird in real flight can be recreated in a wind tunnel setting if the Reynolds number is kept the same. Here, experiments were performed at a Reynolds number of 75,000 which is within the range of bird flight^4^. Flow measurements were conducted at an angle of attack (AoA) of 28 deg which is that which maximized the lift benefit of the alula as per Linehan and Mohseni^19^. The results of these experiments are shown in Fig. 3a,b.

Figure 3a compares streamline flow patterns over the wing with an alula to that of the wing without an alula. Isosurfaces of spanwise velocity are included in the isometric views to help visualize spanwise flow in relation to the LEV. We observe the flow over the top of the wing without an alula to be massively separated, or stalled, as flow stemming from the edges of the wing do not reattach back to the wing. This flow pattern is associated with higher pressure on the top surface of the wing which means a reduction in wing lift.

A very different flow pattern is evident over the wing with an alula in this deep-stall condition. At wing stations outboard of the alula, the flow is reattached near the outer edges of the wing. Here leading- and side-edge streamlines wind around an aft-tilted LEV that smoothly merges with the tip flow forming a recirculatory tip vortex. In contrast, on the adjacent portion of the wing, leading- and side-edge streamlines do not reattach to the wing plane and remain topologically similar to that measured on the wing without the alula. These results show that the alula induces an LEV on the outer wing while the flow over the adjacent wing station remains massively separated.

From an engineering standpoint, it is important to understand how the alula induces and stabilizes the LEV. A helpful way to describe the LEV flow is by analyzing vorticity; a property of the fluid which quantifies its rotation. Figure 3b plots contour slices of the spanwise-oriented (I) and streamwise-oriented (II) components of vorticity on the wing to illustrate the strength of the LEV and tip vortex in these directions. Also included in (I) are contour slices of spanwise velocity to quantify the magnitude of this flow on the wing.

These measurements indicate that the alula induces the LEV by steering separated leading-edge wing flow, in the form of a leading-edge shear layer, back to the wing plane, likely via the interaction of this shear layer with the bottom surface of the alula. Spanwise vorticity accumulates and recirculates to form the LEV as its downstream motion is stymied by an aft-located wall-jet of high magnitude root-to-tip spanwise flow (of magnitude >80% the freestream velocity). This jet-of-flow, produced by the alula, simultaneously tilts LEV vorticity aft and evacuates this flow toward the wing tip via an outboard vorticity flux. We believe these processes facilitate the smooth merging of leading and side-edge vortex flows which in turn stabilizes the resulting vortex system. We contrast the strengthened recirculatory tip vortex on the portion of the wing outboard of the alula with the weak tip vortex on the adjacent wing tip; the latter which more approximately reflects that measured on the wing without the alula. Further details on the mechanisms underlying the alula’s maintenance of the observed attached vortex will appear in a future publication by the authors^23^.

The flow measurements of Fig. 3 represent the first quantitative experimental evidence of a model alula inducing and stabilizing an LEV on the outer portion of an otherwise stalled wing in steady translation. The alula’s ability to maintain this attached vortex preserves an effective bound circulation over the outer portion of the wing and thus sustained lift generation there and thus sustained lift generation there enabling the bird to slow vertical descent and maneuver while airbraking to land. Furthermore, the ability to stabilize the LEV on the spread-wing would enable the bird to max out aerodynamic forces by maximizing wing area.

### Scaling of the alula’s spanwise position on the avian wing

We measured the alula’s spanwise position on spread-wing specimens housed in the Florida Museum of Natural History and on digital spread-wing specimens located in the online digital-image collection of the Slater Museum of Natural History. When combined with existing measurements in the literature^7^, the total data set of bird measurements contains 132 unique species for which 21 of the 36 major orders are represented.

**Figure 4:**
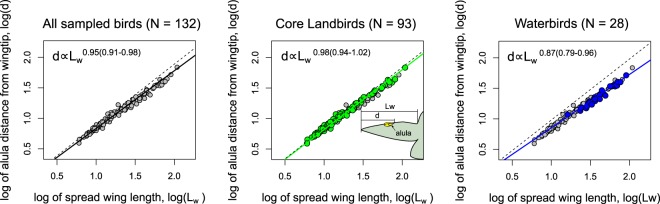
Scaling of the alula’s root distance from wing tip $d$ as a function of spread wing length ${L}_{w}$. Scatter plots of $lo{g}_{10}(d)$ against $lo{g}_{10}({L}_{w})$ for (from left-to-right) all sampled bird species, core landbirds (as per Jarvis *et al*.^24^), and waterbirds (following the ecological scoring of Jarvis *et al*.^24^, Fig. 1). The parentheses denote the 2.5% and 97.5% percentiles of the parameter estimate. The dotted line represents isometric scaling.

**Figure 5:**
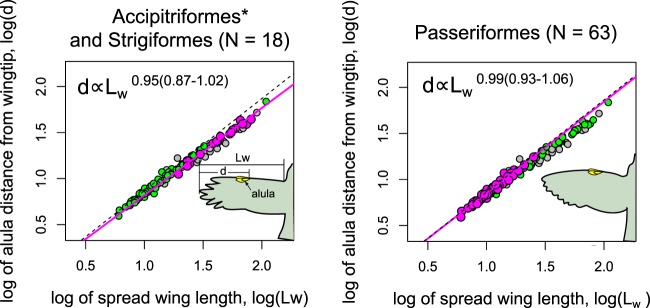
Scaling of the alula’s root distance from the wing tip $d$ as a function of spread wing length ${L}_{w}$ on birds selected for alula optimization. Scatter plots of $lo{g}_{10}(d)$ against $lo{g}_{10}({L}_{w})$ for (from left-to-right) Accipitriformes* and Strigiformes possessing broad-type wings, and Passeriformes possessing elliptical-type wings. The asterisk indicates the removal of *Gyps fulvus*. The parentheses denote the 2.5% and 97.5% percentiles of the parameter estimate. The dotted line represents isometric scaling.

We represent the spanwise position of the alula by $d$ which measures the distance of the alula’s root from the wing tip. The relationship between $d$ and the length of the spread wing ${L}_{w}$ is analyzed using an empirical scaling formula $d=k{L}_{w}^{\alpha }$ where $\alpha$ is the allometric exponent and $k$ is the allometric coefficient. We regress $lo{g}_{10}(d)$ against $lo{g}_{10}({L}_{w})$ to identify deviations in the data from geometric similarity (i.e. $\alpha =1$). When presenting scaling exponents, the parentheses denote the 2.5% and 97.5% percentiles of its estimate. Results are shown in Figs. 4 and 5.

As seen in the results presented in Fig. 4, we found the scaling exponent to be $\alpha =0.95(0.91\mbox{--}0.98)$ when considering all bird species which is statistically below geometric similarity. We found a near isometric scaling ($\alpha =0.98(0.94\mbox{--}1.02)$) when we consider only core landbirds (as per Jarvis *et al*.^24^) and clear allometric scaling when consider birds with aquatic ecologies (labeled waterbirds) ($\alpha =0.87(0.79\mbox{--}0.96)$). The latter waterbird category follows the ecological scoring of Jarvis *et al*.^24^ (Fig. 1 in their work).

We further analyze two groups of birds within core landbirds that possess distinct wing shapes but share selection pressures for alula optimization owing flight in cluttered airspace: Passeriformes which posses an elliptical-type wing and Accipitriformes and Stigiformes which share a broad-type wing (see Fig. 5). The aerial clutter ecological character follows the ‘use of cluttered aispace’ ecological scoring character of Taylor and Thomas^25^. This includes all species in Passeriformes and nearly all species in Accipitriformes and Strigiformes except for *Gyps fulvus* which tends to soar high over clutter^25^. Aerial clutter would require slow flight and high flight forces and thus we expect species that use cluttered airspace to be under selection pressure to maximize the performance of the alula. We found the scaling exponent for Passeriformes to be ($\alpha =0.99(0.93\mbox{--}1.06)$) and that for Accipitriformes and Strigiformes to be ($\alpha =0.95(0.87\mbox{--}1.02)$). The asterisk on Accipitriformes* indicates the removal of *Gyps fulvus*. The approximately linear scaling indicates that trends in the alula’s position for these birds may be described using a single non-dimensional number, the alula’s distance ratio $d/{L}_{w}$.

### Effect of wing shape on the approximate lift-maximizing alula distance ratio

We then performed wind tunnel experiments to approximate the lift-maximizing distance ratio ${(d/{L}_{w})}_{max}$ of model alulae on twelve model wings with shapes and aspect ratios consistent with that of Passeriformes and Accipitriformes and Stigiformes. The elliptical-type wing shape of Passeriformes was modeled using a Zimmerman wing, whereas the broad-type wing shape of Accipitriformes and Stigiformes was modeled using a rectangular wing. The aspect ratio of the model wings ranged from 1.5–4 in increments of 0.5 which encompasses aspect ratio measurements of the half-wing of these birds from Alvarez *et al*.^7^. Experiments were conducted at two Reynolds numbers of 75 K to 100 K which is in the range of bird flight^4^.

In our previous study^19^, we found the surface-oil technique to be a good predictor of the lift-maximal distance ratio of the alula (recall Fig. 2b). In these experiments, the lift-maximal alula distance ratio corresponded to the furthest relative distance of the alula from the wing tip that sustains stable LEV flow on the outer wing (as indicated by the existence of a sweeping separation line in the oil-patterns). Thus, we use the surface-oil technique to approximate the lift-maximal distance ratio on our model wings.

We found that the wing’s angle of attack influenced alula-induced LEV stability for a fixed flight speed. Namely, if the angle of attack was too high or too low the LEV may not be stabilized (as evinced by oil-patterns) for a given alula distance, $d$. These results are consistent with our previous results^19^ which showed that the LEV was only stabilized on the wing over a certain range of post-stall angles of attack. Thus, the surface-oil visualization experiments in this study involved an exploratory process of determining the angle of attack that permitted the alula to be placed the furthest distance from the wing tip which maintained a stable LEV on the outer wing. This distance is denoted as ${d}_{max}$. The assumption is that birds, having a fixed alula position, can adjust angle of attack by tilting/twisting their wings (or adjust sweep angle of hand-wing as discussed in the Discussion section) to ensure the stability of the LEV based on their airspeed. We found this angle of attack to vary from 30 deg to 40 deg for Reynolds numbers of 75 K to 100 K, respectively, while remaining negligibly effected by the wing shapes and aspect ratios considered. Here, a higher angle of attack is needed to stabilize the LEV for the higher flight speed. We provide an explanation of these trends in the Discussion section.

**Figure 6:**
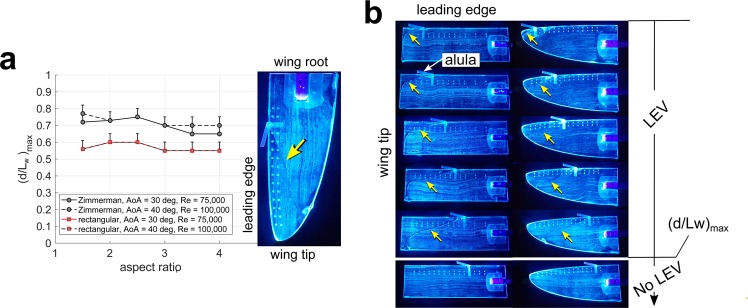
Effect of wing shape on the approximate lift-maximizing relative position of the model bird alula. (**a**) The furthest normalized distance of the alula from the wing tip that maintains a stable LEV, ${(d/{L}_{w})}_{max}$, as a function of aspect ratio, , for Zimmerman and rectangular wings at the test conditions as indicated. (**b**) Representative images of the  = 3 rectangular and Zimmerman wing with an alula displaced increasingly farther from the wing tip. LEV is marked by arrows. The portion of the wing experiencing LEV flow and thus enhanced lift increases in area as the alula is distanced farther from the wing tip until a certain distance for which LEV is lost. ${(d/{L}_{w})}_{max}$, is is as indicated. Angle of attack is 30 deg. Reynolds number is 75,000.

Experimental results for the lift-maximal distance ratio, ${(d/{L}_{w})}_{max}$, for all test cases are plotted in Fig. 6a. A representative set of oil-visualizations used to determine ${(d/{L}_{w})}_{max}$ for the  = 3 rectangular and Zimmerman wing are shown in Fig. 6b where ${(d/{L}_{w})}_{max}$ is marked. We found ${(d/{L}_{w})}_{max}$ to be consistently smaller on rectangular wings than Zimmerman wings. Moreover, we found Reynolds number and aspect ratio (in the tested ranges) to only have a minor influence on ${(d/{L}_{w})}_{max}$, with Reynolds number having no discernible influence for rectangular wings.

The minor variation of ${(d/{L}_{w})}_{max}$ with wing aspect ratio is consistent with our previous finding of a linear relationship between the alula and wing length on sampled birds selected for alula optimization. Namely, if the ${(d/{L}_{w})}_{max}$ were to be dependent on both wing length and aspect ratio then we would expect a nonlinear scaling relationship between $d$ and ${L}_{w}$ on these birds.

### Lift-maximizing alula distance ratio predicts relative alula position on bird wings

We now compare the lift-maximizing distance ratio determined via wind tunnel experiments on model wings to positional measurements on bird wings. If the alula’s relative position is that which maximizes LEV-lift during slow gliding flight, then we would expect the estimate of the distance ratio $d/{L}_{w}$, determined from a linear regression of $d$ against ${L}_{w}$, to equate with the lift-maximal distance ratio ${(d/{L}_{w})}_{max}$ found on model bird wings in a wind tunnel. Furthermore, we would expect trends in ${(d/{L}_{w})}_{max}$ with respect to wing shape to be reflected in $d/{L}_{w}$.

**Figure 7:**
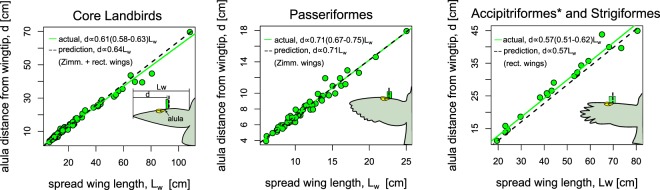
Lift-maximizing alula distance ratio predicts parameter estimates of the alula distance ratio on core landbirds selected for alula optimization. Distance of the alula’s root from the wing tip, $d$, as a function of spread-wing length, ${L}_{w}$, for landbirds, Passeriformes only, and Accipitriformes and Strigiformes only. The parentheses denote the” 2.5% and 97.5% percentiles of the parameter estimate. Schematics compare confidence intervals to the lift-optimal predicted value graphically.

Figure 7 plots measurements $d$ as a function of ${L}_{w}$ for all sampled core landbirds, Passeriformes only, and Accipitriformes* and Strigiformes only. The estimated distance ratio and the 2.5% and 97.5% percentiles of the parameter estimate, as determined via phylogenetic least-squares regression of $d$ on ${L}_{w}$, are labeled as ‘actual’ in the legend. From this comparison, we found $d/{L}_{w}$ to be higher on Passeriformes (0.71(0.67–0.75)) than on Accipitriformes* and Strigiformes (0.57(0.51–0.62)). We note that controlling for phylogeny and branch length assignment had rather little effect on the estimate of the distance ratio, $d/{L}_{w}$, (see Supplementary Information, Fig. S2). For example the estimated distance ratios for core landbirds, Passeriformes only, and Accipitriformes* and Strigiformes only, were 0.60(0.58–0.61), 0.70(0.67–0.74), and 0.56(0.50–0.61) when observations are treated as statistically independent.

The above results are consistent with trends found in wind tunnel experiments regarding the effect of wing shape on the lift-maximal distance ratio, where the elliptical-type Zimmerman wings were found to have notably higher values of ${(d/{L}_{w})}_{max}$ than rectangular wings. To show how well ${(d/{L}_{w})}_{max}$ predicts $d/{L}_{w}$ we add a predictive line (dotted line) to each plot whose slope is computed from averaging ${(d/{L}_{w})}_{max}$ values determined via experiments. For the figure titled core landbirds, the slope of the predicted line is the average of all ${(d/{L}_{w})}_{max}$ plotted in Fig. 6a. For the figure titled Passerines only and Accipitriformes* and Strigiformes only, the slope of the predicted line is the average of all ${(d/{L}_{w})}_{max}$ determined on Zimmerman wings and rectangular wings, respectively.

From Fig. 7, we observe the lift-maximal distance ratio when considering all tested wing models, ${(d/{L}_{w})}_{max}=0.64$, to nearly approximate the alula distance ratio estimated when considering all core landbirds (0.61(0.58–0.63)). Furthermore, we found the lift-maximal distance ratio of the alula determined on Zimmerman wings, ${(d/{L}_{w})}_{max}=0.71$, to approximately equate to the alula distance ratio on sampled Passeriformes (0.71(0.67–0.75)). Moreover, we found the lift-maximal distance ratio of the alula determined on rectangular wings, ${(d/{L}_{w})}_{max}=0.57$, to approximately equate to the alula distance ratio on sampled Accipitriformes* and Strigiformes (0.57(0.51–0.62)). We note here that adding *Gyps fulvus* to the data set, despite not being thought to be under selection pressure for optimizing alula performance due to soaring flight above the canopy, resulted in an alula distance ratio estimate of 0.60(0.55–0.65) for Accipitriformes and Strigiformes which is not statistically different from the lift-maximizing predicted value for the rectangular wing model ${(d/{L}_{w})}_{max}=0.57$.

## Discussion

The goal of this study was to test the hypothesis that the distance of the alula’s root from bird’s wing tip, $d$, relative to wing length, ${L}_{w}$, is that which maximizes LEV-lift when wing is spread and operated in a deep-stall flight condition. Enhanced wing lift via alula-induced LEV flow over their hand-wing would help birds to reduce sink rate and maintain “flight control during slow flight while the spread-wing posture maximizes aerodynamic forces. This scenario would enable birds to airbrake to expedite landing and/or assist maneuvering in cluttered environments.

**Figure 8:**
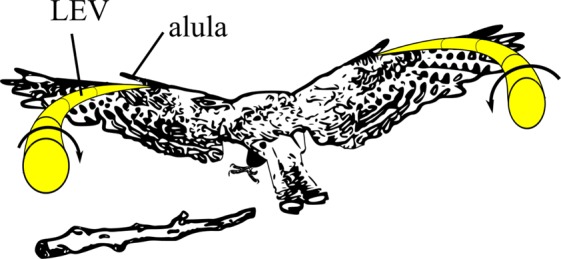
Artist impression of the alula-induced LEVs on the spread wings of a bird executing a glide-assisted landing. The alula is located at the furthest distance from the wing tip that maintains LEV stability on the spread wing which magnifies the high-lift benefit of the alula when birds operate their wings in a deep-stall condition. The ability of the alula to reattach flow on the hand-wing enables the bird to maintain flight control despite the high-angle-of-attack condition. Consequently, large braking forces are generated that enable the bird to land over shorter distances with less landing force.

We found the lift-maximal alula distance ratio, ${(d/{L}_{w})}_{max}$, on elliptical-type model wings and broad-type wings to predict $d/{L}_{w}$ estimates on core landbirds selected for alula optimization. Specifically, ${(d/{L}_{w})}_{max}$ found on Zimmerman wings predicted $d/{L}_{w}$ on Passeriformes and ${(d/{L}_{w})}_{max}$ found on rectangular wings predicted $d/{L}_{w}$ on Accipitriformes* and Strigiformes, respectively. Furthermore, ${(d/{L}_{w})}_{max}$ considering all model wings tested in this study, nearly predicts the $d/{L}_{w}$ estimate on all sampled core landbirds. These results suggest that the relative location of the alula on these birds is that which approximately maximizes alula-induced LEV lift when these birds spread and tilt their wings to post-stall angles to airbrake as necessary to maneuver and land in cluttered environments. A schematic depicting the LEVs induced by bird’s alulae during a glide-assisted landing is shown in Fig. 8. Improved slow-glide performance via the alula-induced LEV can also decrease reliance on flapping during slow flight which would reduce the energy expenditure of the bird. This benefit appears most important for Passeriformes which are generally regarded as active flappers.

In addition to the above findings, we found $d$ on waterbirds to scale more allometrically with ${L}_{w}$ than sampled core landbirds, suggesting that the alula is located closer to the wing tip on waterbirds than core landbirds of like wing length. These results are consistent with Wang and Clarke’s^20^ finding of elongate covert feathers in taxa with aquatic ecologies. We hypothesize that continued take-off and landing in ground effect reduces selection pressures for alula optimization. This may enable aquatic birds to have elongated arm wings that can benefit soaring flight by increasing the cambered airfoil length on the inner wing and thus flight efficiency, in addition to providing the structural rigidity to maintain wing shape to improve glide performance.

We also recalled scaling trends of bird’s primary feathers in the Introduction, noting that primary feather length contributes to the distance of the alula’s root from the wing tip. We can now compare scaling trends between the two. The scaling exponent found in the current study when considering all sampled bird species ($\alpha =0.95(0.91\mbox{--}0.98)$) is not statistically different than the scaling exponent between primary feather length and wing span ($\alpha =0.93$) predicted by Nudds^21,22^. Furthermore, Winkler and Jenni^26^ found the F8 primary feather to encompass 75.5%(73.4–78.2%) of the wing length in 51 Passerine species. This value nearly equates to the apparent lift-maximizing relative alula distance measured on Passeriformes which encompasses (71%(67–75%)) of the wing length. The difference between these two parameters is likely the relative length of the primary covert feathers. We hypothesize that evolutionary adjustments in the length of bird’s primaries could maximize the aerodynamic performance of the alula, and thus the maneuvering and landing performance of the bird, while enabling bird’s arm bones to scale in a manner that satisfies the structural constraints of the wing. A test of this hypothesis might involve a comparative analysis of the relative alula location, arm bone length, and primary feather length that controls for wing shape, bird mass, and ecology.

We also found the angle of attack of the wing to influence the stability of the alula-induced LEV for a fixed flight speed. Namely, if the angle of attack was too high or too low (for a given airspeed) the LEV may not be stabilized for a given alula distance, $d$. Our interpretation of this result is as follows. From our flow measurements (recall Fig. 3), we believe that the stability of the LEV has to do with the smooth merging of leading- and side-edge vortex flows. Facilitating this merging, is the aft-located wall-jet of spanwise flow produced by the alula which tilts and convects LEV vorticity toward the wing tips via an outboard vorticity flux. Working against the outward motion of the LEV, however, is a downstream convective flux of LEV vorticity, of magnitude dependent on angle of attack and airspeed, that drives the downstream motion of the LEV. We believe that past a certain post-stall angle of attack, the LEV gets sufficiently shadowed behind the inclined wing such that the magnitude of streamwise flow is sufficiently small relative to the outboard convective flux imposed by the alula that the LEV is able to sweep across the wing and smoothly merge with the tip flow.

We note that wind tunnel experiments on Zimmerman and rectangular model wings also predicted the difference in $d/{L}_{w}$ between Passeriformes and Accipitriformes* and Strigiformes. This suggests that the apparent lift-maximizing relative position of the alula on the wings of these birds may not be a matter of coincidence. An aerodynamic argument for this difference has to do with leading-edge (planform) curvature, or in the case of the broad-type wing, a lack thereof. A curved leading-edge encourages LEV stability by redirecting freestream flow toward the wing tips thereby facilitating the smooth merging of leading and side-edge vortical flows via an outboard vorticity flux^27^. An aerodynamic rationale for why Passeriformes have their alula placed closer toward their wing root than Accipitriformes and Strigiformes is because their elliptical-type wings have a naturally curved leading-edge planform profile. Additional spanwise flow on the wing produced via leading-edge curvature, in addition to that produced by the alula itself, increases the outboard flux of LEV vorticity enabling the alula to be placed further from the wing tip and still sustain LEV flow on the outer wing. The broad-type wing of Accipitriformes and Strigiformes, lacking an inherent mechanism for spanwise flow generation, must rely on the outboard flux of LEV vorticity produced by the alula alone, thus requiring the alula to be placed closer toward the wing tip to stabilize this flow pattern.

We now attempt to explain the role of the swept-back hand wing on LEV stability. We preface this discussion by reiterating that the lift-maximizing distance ratio of the alula was determined on a spread-wing model. This was intentional, as the placement of the alula here would maximize LEV lift when the wing is spread; the latter providing maximal braking force while the former sustains flight control. However, with the alula at this location the LEV was shown to only be stabilized at a certain post-stall angle of attack. Maintaining this attitude may not be achievable throughout an airbraking maneuver, as a bird may need to adjust trim angle to compensate for changes in airspeed. We propose that varying the sweep angle of the hand-wing can allow the bird to stabilize the alula-induced LEV at higher airspeed (lower angles of attack). This is because a swept-back hand wing can increase spanwise flow production (atop that produced by the alula) facilitating the ability of the alula-induced LEV to smoothly merge with the tip flow at the lower angle of attack. In addition, the sweptback hand wing may be useful for extreme angle-of-attack maneuvers. The surface-oil visualizations of Linehan and Mohseni^19^ (shown in Fig. 2a) depicted a streamwise tilting of the LEV at extreme post-stall angles, namely AoA ≥ 35 deg in the figure. The observed M-shape wing in the pitch-up maneuver of a steppe eagle *Aquila nipalensis* recognized by Carruthers *et al*.^6^ may allow for increased interaction of the streamwise-tilted LEV on the swept-back hand wing and thus enhanced wing lift, pitch-up moment, and drag during this maneuver. Indeed, this is consistent with the hypothesis of Carruthers *et al*. regarding the alula functioning like a strake on delta wing aircraft. From the above arguments, a variable-sweep hand-wing may allow for an alula-induced LEV to be sustained throughout much of the landing sequence of birds.

We now address the limitations of this work both in terms of bird alula measurements and aerodynamic measurements. We start by acknowledging that the conclusions of this work are based on positional measurements of the alula performed on curated spread-wing specimens in addition to measurements on live birds from Alvarez *et al*.^7^. Slight variations between this data with that which would be measured on birds in landing or slow maneuvering flight is to be expected and functional measurements of wing span as well as hand-wing length could reconcile these variations. Further error could be introduced in our parameter estimates due to differences in the resting curvature of dried specimens. In physical specimens, we measured the alula position and wing length leaving the resting curvature of the specimen such as to remain consistent with measurements taken on digitally-imaged specimens. The resting curvature will likely vary from some degree between curators likely causing an increased spread in the data.

In terms of aerodynamic measurements, drawing comparisons between wing models in a controlled environment, such as a wind tunnel, and birds in real flight is tricky and always leaves much to be desired. We note that the wind tunnel experiments were conducted with fixed wings in steady translation and thus extrapolations should be limited to gliding flight. Furthermore, because our interest was in the alula-wing interaction under deep-stall conditions we elected to model the bird’s wing and alula in a manner that incorporates what we believe to be the essential physics of the problem. The key parameters anticipated to influence the wing aerodynamics under these conditions were the wing shape and wing aspect ratio. The influence of wing shape on vortical flows was explained previously. Wing aspect ratio is a parameter that controls the relative influence of tip flows on the aerodynamics of the wing^28^, where wings of low aspect ratio promote leading-edge flow reattachment via tip vortex-induced downwash^27^. In contrast to the apparent influence of wing shape on our results, we found minimal effects of aspect ratio (for the aspect ratio range tested). We attribute this finding to the fact that, at the test conditions considered, the flow is massively separated over the wing section adjacent to that harboring alula-induced LEV flow. In our single wing representation, the wing tip vortex on the adjacent wing tip is more approximately organized into a shear layer than a recirculatory vortex (recall streamline patterns and vorticity contours shown in Fig. 3). The consequence of this flow pattern is reduced downwash on the wing (as indicated by the magnitude of velocity vectors in in Fig. 3) and thus a reduced influence of this adjacent tip flow on the LEV flow outboard of the alula.

Owing to the deep-stall flight condition considered in this study, the effects of airfoil shape, camber, and aerodynamic twist were considered secondary to the planform effects previously mentioned and were not modeled. In the deep-stall flight condition, flow separates at or near the wing’s leading-edge and remains separated regardless of profile shape. Thus, edge flow no longer ‘sees’ the top surface of the wing (recall leading-edge streamlines in Fig. 3a). Following these same arguments, differences in airfoil profile and angle of attack between the bird’s thick-profiled arm wing and thin-profiled hand-wing are not expected to influence such massively separated flow. Positive camber on rotating insect-planforms^29^ has been shown to disrupt LEV formation due to the generation of streamwise vortices. However, the vortex generating and stabilizing mechanisms on rotating wings are different than that on steadily-translating wings due to Coriolis and centripetal accelerations inherent to the former wing motion^30^. Cambered profiles have been shown to improve airfoil performance and increase stall angle on thin, low-aspect-ratio wings at low Reynolds numbers^31^. However, the stall angles (AoAs) for higher aspect-ratio wings in their study ( $\ge$ 1.5, AoAs $\le$ 15 deg for Reynolds number of 60 K and 140 K) remain substantially lower than the angles of attack (AoA) for which the alula-induced LEV was observed (AoA = 30 deg for Reynolds number of 75K and AoA = 40 deg for Reynolds number of 100 K).

The influence of wing-tip slots on our findings, such as that created by the spreading of the primary feathers, remains unclear. As shown by Johansson *et al*.^32^ this spreading results in a multi-cored wing tip vortex both on birds in gliding and flapping flight and may contribute to washout (wing twist) and thus reduce effective angle of attack during high angle of attack flight. The effect of the spreading of wing tip vorticity is likely to influence the alula-induced LEV in some manner. However, its direct influence on LEV stability, and thus the lift-maximizing distance of the alula found in this study, requires additional experimentation to resolve.

Collectively, the findings of this study advance our understanding of the unique ways nature utilizes LEVs to augment flight. To date, LEVs have been implicated in the flight of insects^33–37^, slow-flying bats^38^, hummingbirds^39^, swifts^15,16^, large birds such as geese^40^, slow-flying Passerines^17^, stingrays^41^, fish tails^42^, and auto-rotating seeds^43^. Our results confirm the hypothesis of refs. ^4,6^ that the primary aerodynamic function of bird’s alula is to induce and stabilize LEV flow over their hand-wing during slow flight. The main contribution of this work is the finding that the relative spanwise location of these flight feathers on the avian wing is of aerodynamic importance and for some birds is that which maximizes or at least enhances alula-induced LEV lift during airbraking maneuvers.

## Methods

### Bird specimens and measurements

**Figure 9:**
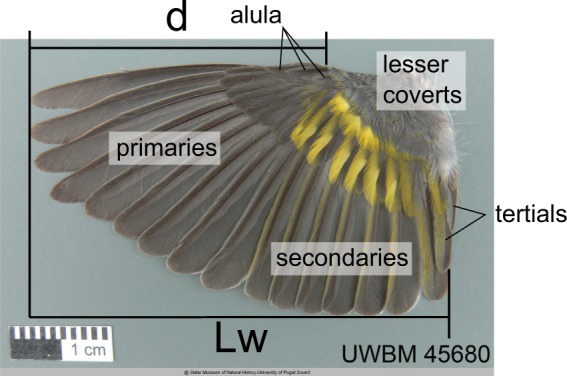
Measurements of the spanwise position of the alula on spread-wing specimens. Definition of $d$ and ${L}_{w}$ from a spread wing specimen. The distance of the alula’s root to the wing tip, $d$, is measured horizontally from the distal end of the longest primary feather to the leading-edge location where the lesser coverts and alula feathers intersect. The spread-wing length, ${L}_{w}$, is the horizontal distance measurement from the distal end of the longest primary feather to the intersection of the proximal edge of the shortest secondary feather with the adjacent tertiary feather.

Measurements were made on spread-wing specimens housed in the Florida Museum of Natural History and on digital spread-wing specimens located in the online digital-image collection of the Slater Museum of Natural History. Figure 9 depicts an example digital specimen and definitions of measured parameters. Here, $d$ is the distance of the alula’s root from the wing tip and ${L}_{w}$ is the spread-wing length. The distance of the alula’s root to the wing tip, $d$, was measured horizontally from the distal end of the longest primary feather to the leading-edge location where the lesser coverts and alula feathers intersect. The spread-wing length, ${L}_{w}$, was the horizontal distance measurement from the distal end of the longest primary feather to the intersection of the proximal edge of the shortest secondary feather with the adjacent tertiary feather. When measuring ${L}_{w}$ on physical specimens, the secondaries and tertials were oriented perpendicular to the wing’s leading edge. Physical measurements were made with a ruler with a corresponding measurement uncertainty of ±0.16 cm. In performing physical measurements we left the resting curvature of the specimen such as to remain consistent with measurements taken on digitally-imaged specimens. A catalog of bird specimens and associated measurements are tabulated in the Supplemental File titled Bird Mesurements. Bird measurements obtained from Alvarez *et al*.^7^ are labeled AlvarezData.

### Statistical analysis

All statistics were controlled for phylogeny. http://birdtree.org/ provides dated phylogenies of the majority of extant bird species for which sets of 100000 psuedo-posterior samples of these phylogenies are available to download. We retrieved 5000 of these trees to represent the possible phylogenetic relationships between the 132 sampled bird species. These trees used Hackett *et al*. 2008 as a backbone. A majority-rule consensus tree (>0.5) was then computed in Mesquite 2.75^44^. Only 123 out of the 132 species were able to be resolved from this tree. Unresolved species were connected to bird species of like family and their branch lengths were equated to neighboring taxa. Lastly, the ultrametricize function in Mesquite was used to adjust branch lengths of terminal taxa. To test the effect of branch lengths on our results we created a second tree for which branch lengths were scaled using the method of Grafen^45^. Both trees were imputed into R and phylogenetic generalized least-squares analysis was performed using the APE package^46^ to assess the relationship between the alula’s root distance from the wingtip and spread wing length. The correlation structure for the function was set to Brownian motion and the model was fit by maximizing the log-likelihood (‘ML’). Comparisons of the phylogenetic least-squares estimate of the alula distance ratio using both trees is shown in the Supplementary Material compared to a non-phylogenetically informed regression analysis. The ultrametric time tree was used for all statistical analysis in the main text.

### Wind tunnel

The wind tunnel set-up has been described in multiple publications by our group^19,47,48^. Experiments were performed in the Engineering Laboratory Design recirculating wind tunnel located at the University of Florida. The test section has a 61 $\times$ 61 cm^2^ cross-section and is 2.44 m in length. The wind tunnel can achieve freestream velocities ranging from 3–91.4 m/s. The turbulence intensity of the freestream was 0.12% at the flow velocities considered.

### Alula models

Each alula was rectangular with a thickness of 0.08 cm, a span of $0.15{L}_{w}$, and an area of $0.01S$ where ${L}_{w}$ is the wing length and $S$ is the wing area. The relative dimensions of the alula considered was motivated by the alula measurements of birds of ref. ^7^. The leading-edge of each alula was offset 0.007c in front of the leading-edge of the wing. The orientation of the alula relative to the wing is defined by three angles: 1. the incidence angle, or the angle of the alula’s chord relative to the wing chord. 2. the deflection angle or cant angle, defined by the rotation of the alula from the plane of the wing about a longitudinal axis aligned with the alula’s root. 3. the pronation angle, or the sweep angle of the alula (in the plane of the wing) relative to the wing’s leading edge. The incidence angle and the sweep angle of the alula were kept at zero degrees whereas the cant angle of the alula was $25$ deg. This value is that which maximized the aerodynamic performance of the model alula in Linehan and Mohseni^19^. Alulae made for the high-aspect-ratio wings were structurally reinforced by gluing a thin metal wire on the back surface of the alula (at the mid-chord location) to reduce static deflection under wind load.

Alula models were printed using a 3D Systems Projet 2500 multijet printer which has been described in ref. ^19^. The printer has a net build volume (XYZ) of 294 × 211 × 144 mm with a 800 × 900 × 790 DPI resolution with 32 $\mu m$ layers. Resolution before post processing is ±0.025–0.05 mm per 25.4 mm of part dimension. The material was VisiJet M2 RWT. Each alula was mounted to the wing via 3D printed, low-profile, press-fit pins.

### Surface-oil flow visualizations

Surface-oil flow visualizations were conducted on a total of twelve wings affixed with an alula (described above). The wings were of rectangular and Zimmerman planform with aspect ratios ranging from 1.5–4 in increments of 0.5. The Zimmerman wing is constructed by mating the major axis of a half ellipse with the minor axis of another half ellipse, where the former half-ellipse forms the wing’s leading edge. Each wing was made of clear acrylic with all edges left square. Thickness-to-chord ratios ranged from was 0.047*c*–0.07*c*. Each wing had mounting holes distanced 0.05${L}_{w}$ across the span of the wing. The mounted holes were drilled with a CNC mill with a tolerance of 0.003 cm. These holes were blind and were drilled from the bottom surface of the wing such as to leave a smooth undisturbed top surface of the wing. Each alula was mounted to each wing via 3D printed, low-profile, press-fit pins.

Tunnel speed was adjusted so that each wing was tested at Reynolds numbers of *Re* = 75,000 and 100,000. Experiments were exploratory in nature and consisted of varying the spanwise position of model alulae and angle of attack to determine the furthest distance of the alula from the wing tip that retained a stable LEV on the outer wing, or ${d}_{max}$. The angle of attack and alula distance were recorded for each Reynolds number.

Surface-oil visualization experiments conducted in this investigation followed a similar procedure as described in ref. ^19^. The oil-pigment mixture consisted of parrafin oil and commercially available fluorescent pigment (Art ‘N Glow pigment powder, particle size 30–50 *μm*). The following procedure was employed: first, a heavily saturated pigment-oil mixture was applied to the inclined wing using a finely bristled brush. The saturated layer was then tipped off with a coarse bristled brush that is wetted with pure parrafin oil. Next, the tunnel velocity was rapidly ramped to the prescribed freestream velocity. After >5 min of run time, the pigment was charged using a UV flashlight and the wing was imaged at inclination with the tunnel still running. Videos of oil-pattern development are available upon request.

### Three-dimensional flow measurements

**Figure 10:**
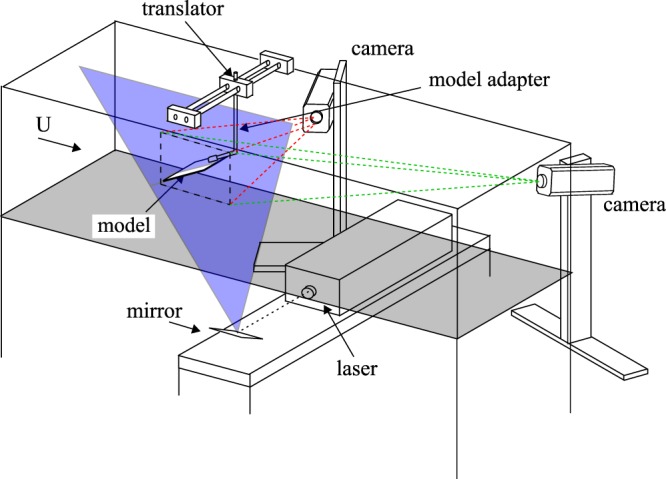
Experimental S-DPIV setup in wind tunnel. The wing-alula model was suspended upside down in the test section via model adapter. Three-component velocity-field measurements were collected in closely-spaced streamwise data planes (2D–3C) by translating the wing-alula model in the cross-stream direction through a stationary vertically-oriented laser plane. These data planes were then time-averaged and subsequently stitched together to grant an approximation of the mean volumetric flow field around the wing-alula model (3D–3C).

A Stereo-Digital Particle Image Velocimetry (S-DPIV) system (see Fig. 10) was used to measure the three-component velocity field in streamwise planes of the flow (2D-3C). The wind tunnel was seeded with ~1 *μ*m olive oil particles generated by an atomizer. These particles were illuminated by a 4 mm thick laser sheet generated by a 20 mJ Nd:YLF laser (Quantronix Darwin Duo, $\lambda =527$ nm). The imaging system consists of high-speed CMOS 1 Mpx cameras (Phantom v210/v211, 1280 × 800 px^2^) with the object-to-image plane mapping function^49^ determined with a precision-machined, dual-plane calibration target. Misalignment of the target with the laser sheet was corrected with the disparity map method^49–51^ for which 100 images (of the undisturbed freestream) were used.

An approximation of the mean volumetric flow field (3D-3C) is constructed from closely-spaced planes of data collected by translating the wing-alula model through a stationary vertically-oriented laser plane. 38 equally-spaced streamwise planes of data were taken a distance $0.06c$ apart on the wing where $c$ is the chord length of the wing. Each plane of S-DPIV data consists of 300 images taken at a rate of 100 Hz (3 sec of acquisition time). Each image was processed with Insight 4G software (ver. 10.0.3.30) by TSI Inc. Images were first dewarped according to calibration images taken for each camera. Thereafter, an iterative multi-pass DPIV evaluation algorithm consisting of windowing shifting/deformation was performed on each image pair. Interrogation windows were made rectangular starting from 40 $\times$ 40 px^2^ down to 20 $\times$ 20 px^2^ (50% overlap). The resulting spatial resolutions of the volumetric flow measurements in the horizontal, vertical, and streamwise directions are Δ$y=0.06c$, Δ*z* = Δ*x* = 0.026*c*. The size of the measurement volume is 2.94c $\times$ 2.28c $\times$ 2.94c where the total number of measured velocity vectors is 113 $\times$ 38 $\times$ 75. A refined grid with four times the resolution, i.e. 452 $\times$ 152 $\times$ 300, is used for the three-dimensional plots.

Measurements were made on a clear acrylic  = 1.5 rectangular wing with and without the alula. The root of the was centrally located on the wing in this test. The chord length of the wing was 9.53 cm, the span length was 14.29 cm. The angle of attack of 28 deg. The thickness of the wing was $0.047c$. All edges were left square. The flow speed was 12.1 m/s corresponding to a Reynolds number of 75,000. 

Statistics of S-DPIV measurements of the undisturbed freestream were used to quantify measurement errors. Taking each time-averaged velocity measurement in space as a single sample, velocity errors corresponding to twice the standard deviation of the sampling distribution were ${e}_{u}/U=0.02$, ${e}_{v}/U=0.01$, and ${e}_{w}/U=0.02$. Vorticity is computed using the local circulation method^52^. An estimate of the error in vorticity from this method is ${e}_{\omega }c/U=0.61{e}_{U}c/(U\Delta x)=0.72$ where *e*_*U*_ is taken as the average of the above velocity errors.

## Supplementary information

Supplementary Information.

Supplementary Information.
