# Supplementary material for: Scaling trends of bird’s alular feathers in connection to leading-edge vortex flow over hand-wing
Source: Sci Rep. 2020 May 13;10:7905. doi: 10.1038/s41598-020-63181-7 (PMC7220954; doi:10.1038/s41598-020-63181-7)
Supplement: Supplementary file 1 — Supplementary Information. [file 41598_2020_63181_MOESM1_ESM.pdf]

Supplementary information of the paper titled “Scaling trends of bird’s alular feathers in connection to leading-edge vortex flow over hand-wing” by Thomas Linehan and Kamran Mohseni

Figure S1: Phylogenetic tree of sampled birds.

Figure S2: Comparison of two trees on the estimate of the alula distance ratio obtained using phylogenetic least squares regression analysis.

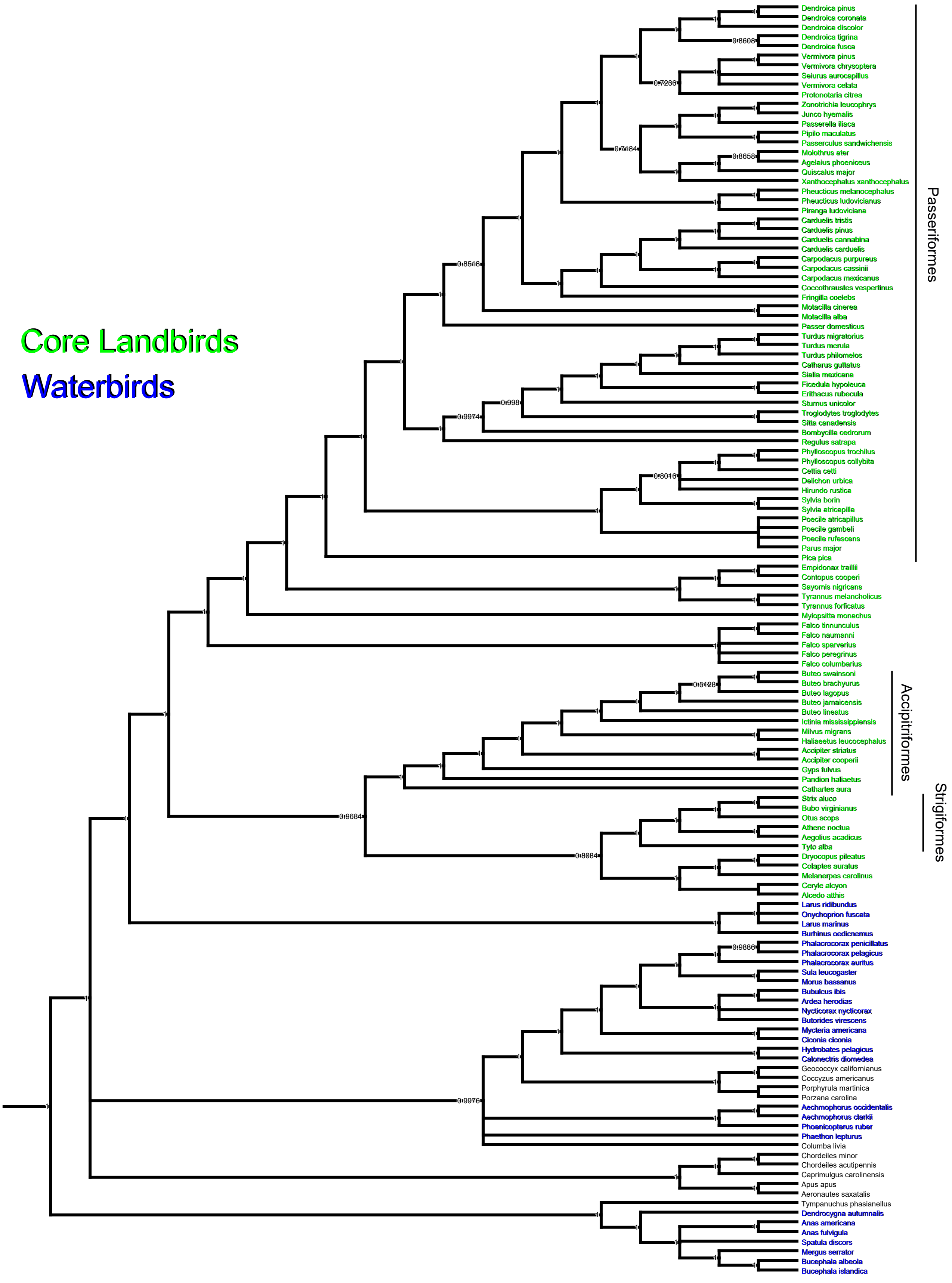

Figure S1 Consensus phylogenetic tree of sampled birds. Core landbirds and waterbirds as indicated by colored text. Passeriformes, Accipitriformes, and Strigiformes are as labeled.

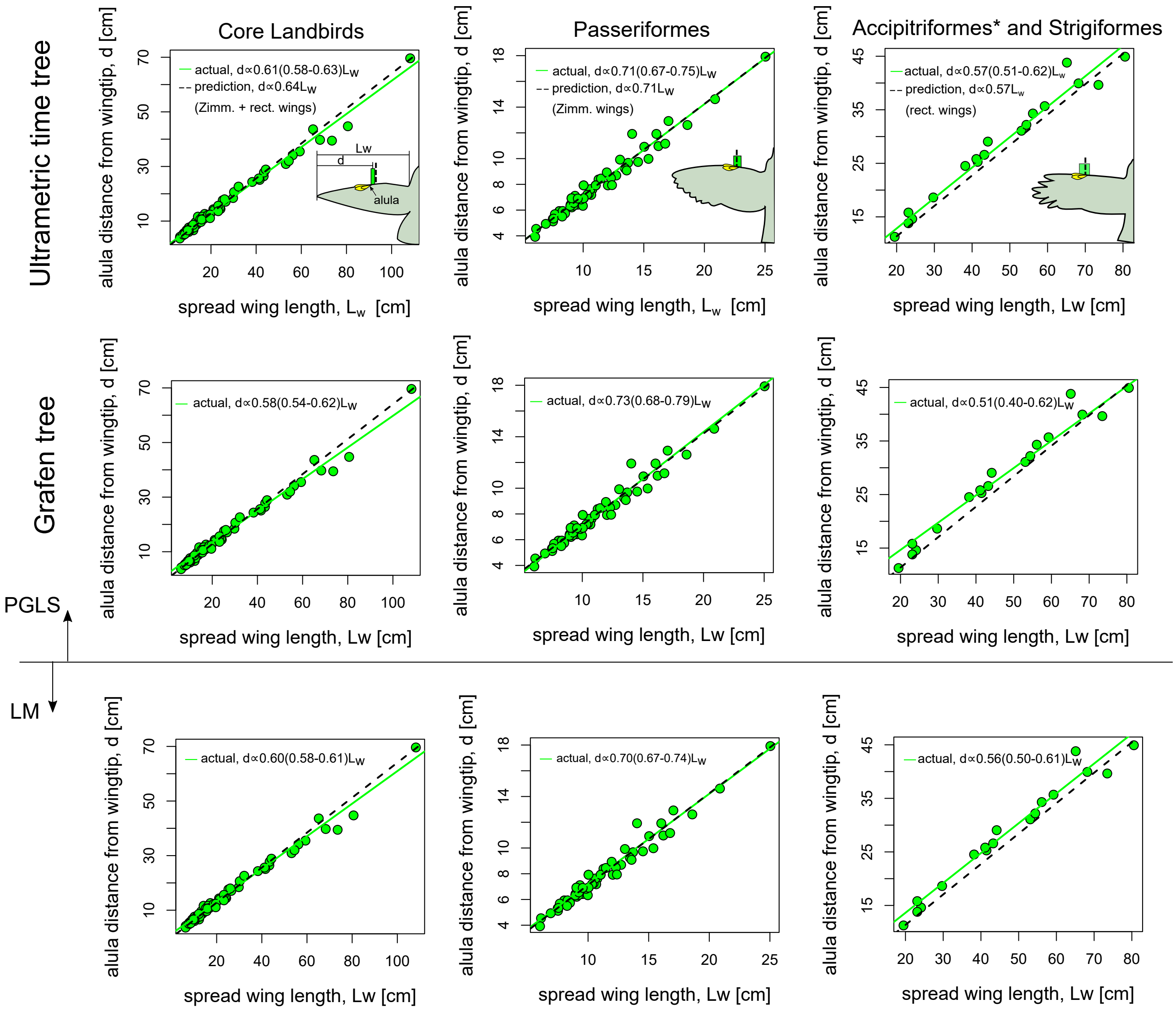

Figure S2 Comparison of two trees on the estimate of the alula distance ratio d/L_w obtained using the phylogenetic least-squares (PGLS) regression analysis. Standard linear model (LM) also shown for reference where observations are treated as statistically independent.
