# Supplementary material for: Scaling trends of bird’s alular feathers in connection to leading-edge vortex flow over hand-wing
Source: Sci Rep. 2020 May 13;10:7905. doi: 10.1038/s41598-020-63181-7 (PMC7220954; doi:10.1038/s41598-020-63181-7)
Supplement: Supplementary file 2 — Supplementary Information. [file 41598_2020_63181_MOESM2_ESM.pdf]

Species	Family	Order	N	Lw.av	Lw.std	d.av	d.std	Catalog
Accipiter cooperii	Accipitridae	Accipitriformes	5	29.6	2.3	18.8	1.1	PSM11269 PSM17148 PSM20846 PSM20958 PSM21589
Accipiter striatus	Accipitridae	Accipitriformes	4	24.0	1.3	14.8	1.1	PSM12144 PSM12194 PSM12340 PSM17142
Buteo jamaicensis	Accipitridae	Accipitriformes	18	53.0	2.8	31.3	3.1	PSM11258 PSM12262 PSM12263 PSM12608 PSM16980 PSM17088 PSM19971 PSM21151 PSM22235 PSM22514 PSM22687 PSM23011 PSM23178 PSM23514 PSM24053 PSM24054 PSM24055 PSM24057
Buteo lagopus	Accipitridae	Accipitriformes	6	59.1	2.5	35.9	1.5	PSM12884 PSM17182 PSM19958 PSM19959 PSM22289 PSM23558
Buteo lineatus	Accipitridae	Accipitriformes	7	41.4	2.4	25.5	1.7	PSM17075 PSM17283 PSM21987 PSM22841 PSM22844 PSM24340 PSM25526
Buteo swainsoni	Accipitridae	Accipitriformes	3	56.0	3.0	34.5	1.3	PSM12510 PSM12511 PSM25986
Haliaeetus leucocephalus	Accipitridae	Accipitriformes	7	80.4	4.0	45.1	1.9	PSM12499 UF41890 UF42299 UF41174 UF41626 UF52443
Ictinia mississippiensis	Accipitridae	Accipitriformes	4	38.1	2.5	24.7	2.7	PSM25419 PSM25987 UWBM45879 UWBM80092
Pandion haliaetus	Accipitridae	Accipitriformes	4	68.1	5.1	40.1	4.2	PSM12471 PSM12714 PSM12936 PSM19963
Milvus migrans	Accipitridae	Accipitriformes	2	65.0	2.0	44.0	0.0	AlvarezData
Gyps fulvus	Accipitridae	Accipitriformes	8	108.0	6.0	70.0	2.0	AlvarezData
Buteo brachyurus	Accipitridae	Accipitriformes	5	43.1	1.5	26.8	0.7	UF45215 UF51213 UF47003 UF46207 UF48707
Cathartes aura	Cathartidae	Accipitriformes	2	73.4	1.1	39.8	3.1	PSM11522
Aechmophorus clarkii	Podicipedidae	Podicipediformes	7	28.3	2.3	16.3	1.3	PSM17241 PSM17243 UWBM42995 UWBM42998 UWBM44677 UWBM54950 UWBM54959

Species	Family	Order	N	Lw.av	Lw.std	d.av	d.std	Catalog
Aechmophorus occidentalis	Podicipedidae	Podicipediformes	8	32.7	1.7	17.5	1.2	PSM12171 PSM12309 PSM12385 PSM12420 PSM12421 PSM21170 PSM21532 PSM21968
Aegolius acadicus	Strigidae	Strigiformes	2	19.4	0.3	11.5	0.4	PSM11477
Bubo virginianus	Strigidae	Strigiformes	6	54.3	2.0	32.4	1.5	PSM11192 PSM11230 PSM12315 PSM12427 PSM12522 PSM12716
Otus scops	Strigidae	Strigiformes	4	23.0	0.0	14.0	0.0	AlvarezData
Athene noctua	Strigidae	Strigiformes	6	23.0	1.0	16.0	0.0	AlvarezData
Strix aluco	Strigidae	Strigiformes	4	41.0	1.0	26.0	1.0	AlvarezData
Tyto alba	Tytonidae	Strigiformes	5	44.0	1.4	29.2	0.6	PSM12155 AlvarezData
Aeronautes saxatalis	Apodidae	Apodiformes	2	15.4	0.6	12.7	0.1	PSM20195
Apus apus	Apodidae	Apodiformes	3	19.0	0.0	17.0	0.0	AlvarezData
Agelaius phoeniceus	Icteridae	Passeriformes	5	14.5	1.0	9.8	0.8	PSM17110 PSM20024 PSM20405 PSM22528 PSM22684
Molothrus ater	Icteridae	Passeriformes	4	13.6	0.3	9.2	0.3	PSM20157 PSM20267 PSM20269 PSM20306
Quiscalus major	Icteridae	Passeriformes	3	20.8	2.8	14.7	2.2	PSM17080 UWBM37121 UWBM45716
Xanthocephalus xanthocephalus	Icteridae	Passeriformes	3	18.6	0.9	12.7	0.5	PSM12670 PSM17242 PSM22239
Bombycilla cedrorum	Bombycillidae	Passeriformes	2	10.7	0.2	7.7	0.0	PSM11217
Contopus cooperi	Tyrannidae	Passeriformes	3	13.4	0.6	9.3	0.2	PSM11320 PSM12008 PSM19911
Empidonax traillii	Tyrannidae	Passeriformes	3	9.2	0.4	6.4	0.5	PSM20498 PSM21248 PSM21360
Sayornis nigricans	Tyrannidae	Passeriformes	2	12.3	0.4	8.0	0.1	UWBM48318
Tyrannus forficatus	Tyrannidae	Passeriformes	2	16.7	0.5	11.2	0.6	UWBM54652
Tyrannus melancholicus	Tyrannidae	Passeriformes	2	15.4	1.0	10.1	0.5	UWBM37780
Dendroica coronata	Parulidae	Passeriformes	8	8.2	0.7	6.0	0.2	PSM11244 PSM20192 PSM20665 PSM21807 PSM21932 PSM22338 PSM22807 PSM22811
Dendroica discolor	Parulidae	Passeriformes	2	7.5	0.1	5.4	0.0	UWBM37154
Dendroica fusca	Parulidae	Passeriformes	2	9.0	0.1	6.3	0.0	UWBM69044
Dendroica pinus	Parulidae	Passeriformes	2	9.3	0.7	7.2	0.5	UWBM42124
Dendroica tigrina	Parulidae	Passeriformes	2	9.5	0.5	6.5	0.0	UWBM41507
Protonotaria citrea	Parulidae	Passeriformes	2	9.2	0.3	6.6	0.1	UWBM40907
Seiurus aurocapillus	Parulidae	Passeriformes	6	9.8	0.5	6.9	0.2	PSM12288 PSM20964 PSM20990 PSM22940 PSM23085 PSM23474

Species	Family	Order	N	Lw.av	Lw.std	d.av	d.std	Catalog
Vermivora celata	Parulidae	Passeriformes	4	7.5	0.0	5.4	0.2	PSM10965 PSM12997 PSM20481 PSM21201
Vermivora chrysoptera	Parulidae	Passeriformes	2	8.5	0.3	5.8	0.2	UWBM45591
Vermivora pinus	Parulidae	Passeriformes	2	8.2	0.4	5.6	0.2	UWBM45603
Carduelis pinus	Fringillidae	Passeriformes	10	9.5	0.4	6.8	0.3	PSM11283 PSM11374 PSM12115 PSM12374 PSM12418 PSM12710 PSM17396 PSM17397 PSM20030 PSM21042
Carduelis tristis	Fringillidae	Passeriformes	4	8.9	0.5	6.5	0.1	PSM12324 PSM17464 PSM20866 PSM21252
Carpodacus cassinii	Fringillidae	Passeriformes	7	11.2	0.4	8.5	0.4	PSM11422 PSM12539 PSM12926 PSM20416 PSM20558 PSM20641 PSM20654
Carpodacus mexicanus	Fringillidae	Passeriformes	5	10.6	0.4	7.3	0.3	PSM20271 PSM20615 PSM20732 PSM20867 PSM21230
Carpodacus purpureus	Fringillidae	Passeriformes	6	10.4	0.5	7.3	0.2	PSM11249 PSM11250 PSM12322 PSM12476 PSM20735 PSM23194
Coccothraustes vespertinus	Fringillidae	Passeriformes	7	13.7	0.8	9.7	0.4	PSM17322 PSM17324 PSM17403 PSM17408 PSM20011 PSM20872 PSM23165
Fringilla coelebs	Fringillidae	Passeriformes	9	11.0	0.0	8.0	0.0	AlvarezData
Carduelis carduelis	Fringillidae	Passeriformes	16	10.0	0.0	7.0	0.0	AlvarezData
Carduelis cannabina	Fringillidae	Passeriformes	2	10.0	0.0	8.0	0.0	AlvarezData
Catharus guttatus	Turdidae	Passeriformes	10	10.6	0.6	7.7	0.5	PSM11204 PSM12393 PSM17381 PSM20666 PSM20741 PSM21832 PSM22417 PSM22814 PSM23379 PSM23829
Sialia mexicana	Turdidae	Passeriformes	3	11.9	1.0	9.0	0.7	PSM20699 PSM20723 PSM23006
Turdus migratorius	Turdidae	Passeriformes	4	16.2	0.5	11.0	0.3	PSM10562 PSM22600 PSM22966 PSM23275
Turdus philomelos	Turdidae	Passeriformes	7	15.0	0.0	11.0	1.0	AlvarezData
Turdus merula	Turdidae	Passeriformes	8	16.0	0.0	12.0	0.0	AlvarezData
Junco hyemalis	Emberizidae	Passeriformes	5	8.9	0.4	6.4	0.1	PSM12188 PSM12540 PSM12838 PSM21665 PSM22141
Passerculus sandwichensis	Emberizidae	Passeriformes	2	9.9	0.1	6.4	0.1	PSM12432

Species	Family	Order	N	Lw.av	Lw.std	d.av	d.std	Catalog
Passerella iliaca	Emberizidae	Passeriformes	3	10.3	0.6	7.3	0.4	PSM10979 PSM22914 PSM23069
Pipilo maculatus	Emberizidae	Passeriformes	8	9.5	0.8	6.9	0.4	PSM12202 PSM12222 PSM12502 PSM20284 PSM20639 PSM20857 PSM20987 PSM21106
Zonotrichia leucophrys	Emberizidae	Passeriformes	3	9.5	0.1	7.0	0.1	PSM20551 PSM21361 PSM23367
Pheucticus ludovicianus	Cardinalidae	Passeriformes	3	12.7	0.5	8.8	0.2	PSM19854 PSM20514 PSM21833
Pheucticus melanocephalus	Cardinalidae	Passeriformes	3	12.3	0.3	8.6	0.2	PSM20848 PSM20875 PSM21374
Piranga ludoviciana	Thraupidae	Passeriformes	3	11.4	0.1	8.5	0.2	PSM10955 PSM17345 PSM20148
Poecile atricapillus	Paridae	Passeriformes	5	7.5	0.7	5.2	0.4	PSM11360 PSM12989 PSM17388 PSM21118 PSM21231
Poecile gambeli	Paridae	Passeriformes	4	7.6	0.4	5.8	0.3	PSM20414 PSM20453 PSM20745 PSM21114
Poecile rufescens	Paridae	Passeriformes	7	6.9	0.4	5.0	0.3	PSM12182 PSM12402 PSM12517 PSM17156 PSM20640 PSM21365 PSM21649
Parus major	Paridae	Passeriformes	9	10.0	0.0	7.0	0.0	AlvarezData
Regulus satrapa	Regulidae	Passeriformes	3	6.1	0.4	4.6	0.1	PSM11259 PSM12181 PSM12190
Sitta canadensis	Sittidae	Passeriformes	5	8.3	0.5	6.0	0.3	PSM12139 PSM12967 PSM20566 PSM20935 PSM22803
Hirundo rustica	Hirundinidae	Passeriformes	6	14.0	0.0	12.0	0.0	AlvarezData
Delichon urbica	Hirundinidae	Passeriformes	5	13.0	0.0	10.0	0.0	AlvarezData
Motacilla cinerea	Motacillidae	Passeriformes	8	11.0	0.0	8.0	0.0	AlvarezData
Motacilla alba	Motacillidae	Passeriformes	19	12.0	0.0	8.0	1.0	AlvarezData
Troglodytes troglodytes	Troglodytidae	Passeriformes	6	6.0	0.0	4.0	0.0	AlvarezData
Erithacus rubecula	Muscicapidae	Passeriformes	9	9.0	0.0	7.0	0.0	AlvarezData
Ficedula hypoleuca	Muscicapidae	Passeriformes	3	10.0	0.0	8.0	0.0	AlvarezData
Cettia cetti	Cettiidae	Passeriformes	5	8.0	0.0	6.0	0.0	AlvarezData
Sylvia borin	Sylviidae	Passeriformes	8	10.0	1.0	7.0	0.0	AlvarezData
Sylvia atricapilla	Sylviidae	Passeriformes	16	9.0	0.0	7.0	0.0	AlvarezData
Phylloscopus collybita	Phylloscopidae	Passeriformes	27	8.0	0.0	6.0	0.0	AlvarezData
Phylloscopus trochilus	Phylloscopidae	Passeriformes	36	8.0	0.0	6.0	0.0	AlvarezData
Pica pica	Corvidae	Passeriformes	6	25.0	1.0	18.0	0.0	AlvarezData
Sturnus unicolor	Sturnidae	Passeriformes	4	17.0	0.0	13.0	0.0	AlvarezData
Passer domesticus	Passeridae	Passeriformes	6	10.0	0.0	7.0	0.0	AlvarezData

Species	Family	Order	N	Lw.av	Lw.std	d.av	d.std	Catalog
Anas americana	Anatidae	Anseriformes	5	35.5	4.2	22.4	2.3	PSM10942 PSM21070 PSM21425 PSM21856 PSM24400
Bucephala albeola	Anatidae	Anseriformes	4	23.3	0.7	13.9	0.7	PSM11262 PSM54041 UWBM65203 UWBM79711
Bucephala islandica	Anatidae	Anseriformes	19	29.0	2.7	18.3	1.8	PSM22601 PSM24072 UWBM48042 UWBM51933 UWBM51934 UWBM51936 UWBM51937 UWBM51939 UWBM51941 UWBM51942 UWBM51943 UWBM51944 UWBM51945 UWBM51946 UWBM52081 UWBM58527 UWBM58528 UWBM63730 UWBM63731
Anas fulvigula	Anatidae	Anseriformes	5	30.7	2.3	19.8	1.2	UF50049 UF47078 UF40728 UF21959 UF48716
Dendrocygna autumnalis	Anatidae	Anseriformes	3	32.8	1.2	19.4	0.7	UF51205 UF45220 UF49930
Mergus serrator	Anatidae	Anseriformes	5	30.3	1.5	18.6	0.9	UF47069 UF45721 UF44941 UF42306 UF46965
Spatula discors	Anatidae	Anseriformes	5	23.9	1.4	15.6	0.5	UF42823 UF48125 UF47081 UF42823 UF43818
Ardea herodias	Ardeidae	Ciconiiformes	4	64.8	7.3	34.9	5.1	PSM11426 PSM12762 PSM16965 PSM19965
Butorides virescens	Ardeidae	Ciconiiformes	5	27.7	2.8	15.3	1.7	PSM11189 PSM12277 PSM12945 PSM20538 PSM22444
Nycticorax nycticorax	Ardeidae	Ciconiiformes	3	47.1	0.7	25.7	1.7	PSM12023 PSM12024 PSM19952
Mycteria americana	Ciconiidae	Ciconiiformes	8	68.8	2.7	35.9	1.3	UWBM56999 UF45478 UF44044 UF46854 UF40986 UF40591 UF44049 UF40989
Ciconia ciconia	Ciconiidae	Ciconiiformes	10	90.0	3.0	53.0	2.0	AlvarezData
Ceryle alcyon	Alcedinidae	Coraciiformes	2	22.3	0.4	14.3	0.1	PSM21407
Alcedo atthis	Alcedinidae	Coraciiformes	4	12.0	0.0	7.0	0.0	AlvarezData
Chordeiles acutipennis	Caprimulgidae	Caprimulgiformes	4	24.8	0.7	15.9	1.0	PSM23093 PSM23848 PSM24548 PSM24549
Chordeiles minor	Caprimulgidae	Caprimulgiformes	3	26.5	0.2	16.6	0.1	PSM12661 PSM20400 PSM23534

Species	Family	Order	N	Lw.av	Lw.std	d.av	d.std	Catalog
Caprimulgus carolinensis	Caprimulgidae	Caprimulgiformes	5	27.0	0.6	17.9	0.8	UF43040 UF41402 UF44156 UF43092 UF43791
Coccyzus americanus	Cuculidae	Cuculiformes	7	17.0	0.7	12.2	0.4	PSM22930 UF42843 UF42345 UF42149 UF43849 UF21895
Geococcyx californianus	Cuculidae	Cuculiformes	4	20.1	0.7	13.7	0.4	PSM23009 UF47269 UF47523
Colaptes auratus	Picidae	Piciformes	11	21.1	0.9	14.3	0.5	PSM17128 PSM17487 PSM20502 PSM20535 PSM21588 PSM21885 PSM21997 PSM22935 PSM24110 PSM24478 PSM25853
Dryocopus pileatus	Picidae	Piciformes	5	25.8	1.0	18.4	0.6	UF48464 UF43850 UF44032 UF47089 UF47089
Melanerpes carolinus	Picidae	Piciformes	6	15.7	1.2	11.0	0.6	UF44054 UF43922 UF43904 UF43923 UF42160 UF41805
Columba livia	Columbidae	Columbiformes	12	29.1	1.1	20.8	1.1	PSM11000 PSM12609 PSM17193 PSM17428 PSM20721 PSM20879 PSM23872 AlvarezData
Falco columbarius	Falconidae	Falconiformes	9	26.3	1.9	17.5	1.0	PSM12757 PSM17183 PSM20465 PSM21093 PSM22462 PSM22583 PSM22726 PSM23504 PSM24044
Falco peregrinus	Falconidae	Falconiformes	4	43.2	3.9	28.2	2.4	PSM19885 PSM21186 PSM22483 PSM22661
Falco sparverius	Falconidae	Falconiformes	6	23.7	2.6	15.2	1.7	PSM12658 PSM12674 PSM17346 PSM17424 PSM21010 PSM23383
Falco naumanni	Falconidae	Falconiformes	13	30.0	1.0	21.0	2.0	AlvarezData
Falco tinnunculus	Falconidae	Falconiformes	4	32.0	1.0	23.0	1.0	AlvarezData
Morus bassanus	Sulidae	Pelecaniformes	2	69.9	3.4	41.3	2.0	PSM22836
Sula leucogaster	Sulidae	Pelecaniformes	3	58.3	2.4	34.8	1.7	PSM21828 PSM21829 PSM21993
Phalacrocorax auritus	Phalacrocoracidae	Pelecaniformes	3	48.8	1.4	24.6	1.1	PSM16982 PSM17186 PSM21083
Phalacrocorax pelagicus	Phalacrocoracidae	Pelecaniformes	5	35.9	2.0	20.0	0.9	PSM11460 PSM12056 PSM12057 PSM12787 PSM21576
Phalacrocorax penicillatus	Phalacrocoracidae	Pelecaniformes	4	41.4	3.0	21.6	1.0	PSM20433 PSM20602 PSM20603 PSM21160
Bubulcus ibis	Ardeidae	Pelecaniformes	13	41.0	1.0	25.0	0.0	AlvarezData
Phoenicopterus ruber	Phoenicopteridae	Phoenicopteriformes	16	75.9	3.8	40.9	1.9	PSM10903 AlvarezData
Porzana carolina	Rallidae	Gruiformes	4	14.7	0.8	8.9	0.6	PSM11466 PSM20856 PSM21032 PSM24385

Species	Family	Order	N	Lw.av	Lw.std	d.av	d.std	Catalog
Porphyrula martinica	Rallidae	Gruiformes	6	20.8	1.0	13.6	0.9	UF44147 UF42417 UF42357 UF44146 UF44518 UF40815
Calonectris diomedea	Procellariidae	Procellariiformes	14	56.0	2.0	35.0	0.0	AlvarezData
Hydrobates pelagicus	Hydrobatidae	Procellariiformes	10	16.0	0.0	12.0	0.0	AlvarezData
Burhinus oedicnemus	Burhinidae	Charadriiformes	2	35.0	0.0	22.0	1.0	AlvarezData
Larus ridibundus	Laridae	Charadriiformes	11	44.0	2.0	30.0	1.0	AlvarezData
Larus marinus	Laridae	Charadriiformes	5	63.2	1.5	37.0	0.9	UF50854 UF45243 UF47843 UF44407 UF52646
Onychoprion fuscata	Laridae	Charadriiformes	5	35.7	0.8	23.2	0.7	UF48507 UF45543 UF50057 UF51817 UF42810
Tympanuchus phasianellus	Phasianidae	Galliformes	2	25.5	2.0	16.7	0.4	UF39695
Myiopsitta monachus	Psittacidae	Psittaciformes	5	19.4	0.4	12.8	0.4	UF40242 UF40237 UF40240 UF43856 UF43926
Phaethon lepturus	Phaethontidae	Phaethontiformes	6	35.8	2.2	21.7	1.9	UF42503 UF43635 UF40589 UF46845 UF44320 UF44505
